# A participatory study of college students’ mental health during the first year of the COVID-19 pandemic

**DOI:** 10.3389/fpubh.2023.1116865

**Published:** 2023-03-21

**Authors:** Chulwoo Park, Melissa McClure Fuller, Thea Marie Echevarria, Kim Nguyen, Daisy Perez, Hufsa Masood, Tasneem Alsharif, Miranda Worthen

**Affiliations:** Department of Public Health and Recreation, San José State University, San José, CA, United States

**Keywords:** course-based undergraduate research experience, depression, anxiety, loneliness, COVID-19, participatory research

## Abstract

**Introduction:**

The COVID-19 pandemic has negatively impacted college students’ mental health and wellbeing. Even before the pandemic, young adults reported high mental health morbidity. During the pandemic, young adult college students faced unprecedented challenges, including campus closure and a pivot to fully online education.

**Methods:**

This study employed a novel participatory approach to a Course-based Undergraduate Research Experience (CURE) in an introductory epidemiology course to examine factors students considered important regarding their experience during the pandemic. Two groups of undergraduate students enrolled in this course (one in Fall 2020 and another in Spring 2021) and participated in the CURE. A sub-group of these students continued after the class and are authors of this article. Through repeated cross-sectional surveys of college students’ peer groups in northern California in October 2020 and March 2021, this student/faculty collaborative research team evaluated depression, anxiety, suicidal ideation and several other topics related to mental health among the students’ young adult community.

**Results:**

There was a high prevalence of anxiety (38.07% in October 2020 and 40.65% in March 2021), depression (29.85% in October 2020 and 27.57% in March 2021), and suicidal ideation (15.94% in October 2020 and 16.04% in March 2021). In addition, we identified the significant burden of loneliness for college students, with 58.06% of students reporting feeling lonely at least several days in the past two weeks. Strategies that students used to cope with the pandemic included watching shows, listening to music, or playing video games (69.01%), sleeping (56.70%), taking breaks (51.65%), and connecting with friends (52.31%) or family (51.21%). Many reported distressing household experiences: more than a third reporting loss of a job or income (34.27%) in the first year of the pandemic. We explain the participatory research approach and share empirical results of these studies.

**Discussion:**

We found this participatory CURE approach led to novel, experience-based research questions; increased student motivation; real-world benefits such as combatting imposter syndrome and supporting graduate school intentions; integration of teaching, research, and service; and development of stronger student-faculty relationships. We close with recommendations to support student wellbeing and promote student engagement in research.

## Introduction

The global COVID-19 pandemic has been devastating for many communities. In the United States, while young adults have had lower mortality rates from COVID-19 than older adults, this age group has experienced massive disruptions to their education, living situations, and livelihoods. Throughout the pandemic, young adults have consistently reported the highest levels of depression and anxiety of any age group (1) and experienced the highest unemployment rates of any age group (2).

Young adult college students have faced unprecedented challenges, including campus closure and a pivot to fully online education. National surveys of college students from March through May 2020, at the start of the pandemic, showed that two-thirds of college students were very or extremely concerned about how long the pandemic would last, and a similar proportion experienced an increase in financial stress (3). In surveys of California college students, a majority reported that they changed their living situation, nearly half lost work, and 40% took on household caregiving responsibilities (4). Over 70% of California college students reported missing class because of personal stress (4).

According to a large, multi-campus study in the summer of 2020, first generation college students were more likely than continuing generation college students to experience financial hardships, food and housing insecurity, encounter technological barriers to online education, and to experience adverse mental health outcomes (5). Black, Latinx, and low-income students were also more likely to experience financial hardships, increased caregiving responsibilities, and have inadequate access to technology to support online learning (4).

While these burdens have been well-documented in a series of reports and articles, little research has been conducted by and for diverse college students, centering students’ concerns about their experiences during the pandemic. For example, although previous research measured mental health among diverse college students in Israel (6) and the United States (7), these studies did not emphasize students engagement in the study design and data collection, or provide an opportunity for students to draw on their experiential knowledge of mental health to generate research questions. Such participatory research has the potential to identify new aspects of wellbeing that have not been previously described, through engaging students to reflect on their experiences in a way that supports meaning-making and purposeful action for improving student wellbeing. The present study aims to fill this gap by describing a Course-based Undergraduate Research Experience (CURE) to conduct participatory research on college student wellbeing during the COVID-19 pandemic at a large, diverse public university in northern California.

This article employs a novel structure: while presenting findings from an empirical epidemiologic study, it documents the participatory methods and presents qualitative evaluative comments from members of the collaborative research team. In addition, using a participatory CURE approach, we illustrate the impacts of conducting this epidemiology research. We share the empirical results of this study which, though limited in their generalizability, produce valid estimates of the burden of various mental health challenges in the peer group of the student research partners. We argue that participatory CURE approaches are an under-utilized methodology in public health and social science disciplines. We encourage more wide-spread adoption focusing on adolescent mental health in school and university settings.

### Participatory course-based undergraduate research experience

CUREs are a form of experiential learning where students gain hands-on research experience within a credit-bearing course. CUREs share five key attributes: they (1) engage students in scientific research, (2) emphasize collaboration, (3) produce new knowledge, (4) focus on broadly relevant topics, and (5) are scaffolded or iterative, allowing for multiple learning opportunities (8–10). While originally promoted in STEM education as a way to scale student involvement in research (11, 12), CUREs have been increasingly recognized as high impact practices for diverse college students to increase retention, promote a sense of belonging in higher education, and enhance diversity in the academic pipeline (9). Empirical research is scant in social sciences and public health on the use of CUREs (10).

Because of their emphasis on engagement, focus on topics relevant to students, orientation toward students as collaborators, and scaffolded learning opportunities, CUREs are a natural fit for participatory research methodologies. Participatory methodologies have been employed in fields as diverse as education, international development, and public health for the past five decades. While the specific methods used in these fields vary, they share a common approach, centering partnership, mutual learning, application/action, and real world impact (13). Within the field of public health, the most commonly used participatory research approaches are Community-Based Participatory Research (CBPR), Participatory Action Research (PAR), and Youth Participatory Action Research (YPAR) (14).

Participatory research methodologies are collaborative and seek to equitably involve academic and non-academic research partners through all phases of a study, from identification of a problem to research design and implementation to analyzing and disseminating the study results. Many participatory studies aim to improve health and health equity (15). Participatory methodologies build on the resources and strengths of community members or participants, valuing lived experience in addition to other forms of knowledge. These methodologies often result in context-specific research and action projects. In addition, participatory methods often use multiple strategies for dissemination of knowledge produced through a study, including forms that are most accessible to community members as well as more traditional academic products or policy/advocacy reports (16).

The present study employed a participatory approach to a CURE study within a public health course. We considered college students as the participants or community of interest, situating the faculty member teaching the course as the “academic partner,” recognizing that, in reality, often participatory research partners occupy multiple, complex positions within a study (17). The CURE design encouraged student co-researchers to draw on the knowledge gained not only in their academic studies, but also from their lived experiences as members of the affected population. In addition, this participatory CURE provided an opportunity for continued involvement in this study after the course had concluded. Indeed, student co-researchers participated in all aspects of the study, including the writing of this manuscript. While all authors participated in the research and writing, we chose to italicize reflections of individual research team members. While there is rich history to this multi-voiced approach in qualitative research (18), this approach is less common in quantitative public health research, and we are not aware of any CURE studies that incorporate student voice as explicitly. We hope that this innovative approach deepens the reader’s experience in learning about this participatory course-based study and provides context to the experience of college students during the COVID-19 pandemic.

## Materials and methods

### Study design

The study took place at a large, diverse, urban public university in northern California. Two linked cross-sectional studies were designed, implemented, and analyzed by college students as part of a CURE embedded in a required junior-level public health introductory epidemiology course. The course instructor (last author) encouraged students to think about their own lives, what they were learning about the pandemic through their classes, news and social media, and the experiences and concerns of their family and friends in order to identify topics that they wanted to explore through a survey.

Working in small groups, students discussed what topics they were generally interested in and then conducted literature reviews to identify what was already known on the topics they selected. With guidance from the instructor, students then developed survey questions on their topic, which were integrated into a single survey assessing their peers’ experiences during the COVID-19 pandemic. When topics examined constructs where standardized scales were available (e.g., depression), the instructor encouraged students to use these standard scales; when topics were novel or no prior scale could be found, original survey questions were developed based on student experiences and perspectives.

The present article describes the work of two different classes, with a focus on results related broadly to mental health. In the fall semester, the class had 25 students working in five teams; in the spring semester, the class had 26 students working in six teams. Each class produced one survey, which pulled together the research questions developed by each of the student teams: one survey conducted during fall semester of 2020 (“October 2020 survey”) and one survey conducted during spring semester of 2021 (“March 2021 survey”). Topics fell broadly under the heading of wellness during COVID-19. Both surveys assessed demographics and mental health. In this pre-vaccine era, students in the fall semester also examined attitudes toward COVID-19 vaccination, employment characteristics, access to personal protective equipment and social distancing at work, and coping strategies that participants were using to deal with the pandemic. In the spring, students added questions focusing on different aspects of mental health, food insecurity, adverse household experiences during the pandemic, as well as use of legal and illicit drugs. The rest of this article focuses on the survey constructs related to mental health. Both study protocols were written by students, edited by the faculty member, and approved by the University’s Institutional Review Board.

The student-faculty collaboration continued after the class ended with five undergraduate students (authors 3–7) joining the instructor, a colleague (first author), and a graduate student (second author) in further data analysis and dissemination. These continuing students led a process where they identified which findings they thought were important to share with the college community and their peers and disseminated these findings to university stakeholders through on-campus presentations, email to the director of the campus health center and other campus leaders, and through a blog.^1^ The study team also collaborated on developing conference abstracts, which they presented in November 2021 at the American Public Health Association annual meeting, and in writing the present article.

### Data collection

Both surveys employed a non-probability sampling design, disseminating a link to an anonymous online Qualtrics^xm^ (Qualtrics International Inc., Provo, UT) survey through email and social media. The October 2020 survey was opened on October 14, 2020 and closed on November 4, 2020; the March 2021 survey was opened on March 23, 2021 and closed on April 13, 2021.

Each class developed their own sampling strategy and inclusion/exclusion criteria. For the October 2020 survey, the class decided to include all adults over the age of 18 who were California residents. This decision was made because many of the students were in their first semester at the university after transferring from community college and students were concerned that if they restricted participants to college students at their university, they might not be able to obtain a sufficiently large sample to examine the questions of interest. However, in documenting where they distributed the survey link, it was apparent that most people who received the survey link were contacted through campus lists (e.g., class lists, student organizations and clubs, and sports teams) and the sample was predominantly college students. For the March 2021 survey, the class decided to only include students over the age of 18 at the university where the study took place. Similar strategies were employed to disseminate the survey link.

### Measures

Both surveys began by assessing the eligibility criteria. If a participant did not meet either of the criteria, they were taken to the end of the survey. If a participant met the criteria, they were asked a series of demographic questions including their age, gender identity, sexual orientation, and racial or ethnic identity.

Both surveys used the Patient Health Questionnaire-4 (PHQ-4) to assess depression and anxiety. These two constructs were coded according to the scale conventions with a total score ≥ 3 for questions 1 and 2 suggesting anxiety and a total score ≥ 3 for questions 3 and 4 suggesting depression (19). The PHQ-4 consists of a 2-item depression scale (PHQ-2) and a 2-item anxiety scale (GAD-2) and has good psychometric properties to measure depression and anxiety in the general population (19, 20).

Both surveys included item 9 of the PHQ-9, which assesses thoughts of self-harm or suicidal ideation. This single item has been found to be a strong predictor of future suicide attempt or completion in large, population-based studies (21, 22). Consistent with other studies of college student mental health, we used this single item as a proxy for suicidal ideation (23–25). We classified responses of “not at all” to this question as not having suicidal ideation and responses of “several days” or more frequently as having suicidal ideation to make it a binary variable.

The October 2020 survey assessed experiences of loneliness by asking how often participants felt lonely or isolated. This question was modified from the CES-D (26), which uses a reference time of 7 days, to use the same 2 week time frame as the PHQ-4 and PHQ-9 item 9. In addition, the survey asked “What are some of the ways you are coping with the COVID-19 pandemic?” and offered the following options: Taking breaks; Sleeping; Mindfulness practice or breathing exercises; Using alcohol or cannabis; Using other drugs; Exercise or spending time outdoors; Art, music, journaling, or another creative expression; Connecting with friends; Connecting with family; Watching shows, listening to music, or playing video games; Taking care of a pet; or Other (specify).

The March 2021 survey asked participants “Overall, how would you say your mental health has been since the start of the pandemic?” with the options of reporting that their mental health had gotten worse, stayed the same, gotten better, or that they were unsure. For participants who reported their mental health had gotten worse, they were asked the follow up question “Do you believe that your worsening mental health is due to the pandemic?” with options to state “Yes, largely due to the pandemic and its associated challenges,” “Somewhat, the pandemic has contributed, but there are other factors, too,” “No, my experience is not really because of the pandemic,” or “Unsure.” In addition, this survey asked participants “In the last 2 weeks, how often have you felt that you were on top of things?” with response options mirroring those available for the PHQ survey items. The March 2021 survey also assessed whether participants or members of their household had any of several distressing experiences during the COVID-19 pandemic.

### Statistical methods

First, we described the demographic characteristics of the population. We then estimated the overall prevalence of each primary outcome and the distribution of these outcomes by demographic characteristic, testing for differences. Second, we assessed differences in the level of mental health pathology (anxiety, depression, and suicidal ideation) across the two time periods by using a qui-square test. We used generalizable linear regression models with robust standard errors to examine hypothesized relationships between variables. All analyses were performed using Stata/MP 14.2 (StataCorp, College Station, TX).

## Results

### Study findings

There were 457 and 245 survey responses, respectively, in October 2020 and March 2021. After excluding respondents who did not meet eligibility criteria or who did not provide answers to any of the survey questions after opening the survey, we were left with analytic samples of 394 participants in October 2020 and 222 participants in March 2021, resulting in a total analytic sample of 616. Demographic characteristics of the samples are provided in Table 1 and show similar distributions by gender, sexual orientation, and race across the two different time frames. More than 70% of participants were female, three-fourths were heterosexual, the majority were Asian or Latinx, and ~75% were aged 18–25.

**Table 1:** Mental health status during COVID-19.

	Total		Anxiety		Depression		Suicidal ideation	
	T1	T2	T1	T2	T1	T2	T1	T2
Characteristic	*n* (%)	*n* (%)	*n* (%)	*n* (%)	*n* (%)	*n* (%)	*n* (%)	*n* (%)
Total	394 (100)	222 (100)	150 (38.07)	87 (40.65)	117 (29.85)	59 (27.57)	62 (15.94)	34 (16.04)
Gender			*p* < 0.001	*p* = 0.003	*p* = 0.089	*p* = 0.415	*p* = 0.024	*p* = 0.306
Man	100 (25.38)	44 (19.82)	20 (20)***	9 (20.45)**	21 (21)	10 (22.73)	16 (16)*	4 (9.1)
Woman	289 (73.35)	174 (78.38)	127 (43.94)***	78 (44.83)**	94 (32.53)	49 (28.16)	43 (14.88)*	29 (16.67)
Other	5 (1.27)	4 (1.80)	3 (60)***	0 (0)**	2 (40)	0 (0)	3 (60)*	1 (25)
Sexual orientation			*p* = 0.002	*p* = 0.789	*p* < 0.001	*p* = 0.614	*p* < 0.001	*p* = 0.520
Heterosexual	322 (82.56)	175 (79.55)	112 (34.78)**	69 (39.43)	79 (24.53)***	46 (26.29)	38 (11.8)***	26 (14.86)
Not heterosexual	68 (17.44)	45 (20.45)	37 (54.41)**	18 (40)	38 (55.88)***	13 (28.89)	23 (33.82)***	8 (17.78)
Age			*p* = 0.153	*p* = 0.655	*p* = 0.168	*p* = 0.161	*P* = 0.027	*p* = 0.146
18–25	297 (75.38)	188 (85.07)	119 (40.07)	75 (39.89)	94 (31.65)	53 (28.19)	54 (18.18)*	32 (17.02)
26 and older	97 (24.62)	33 (14.93)	31 (31.96)	11 (33.33)	23 (23.71)	5 (15.15)	8 (8.25)*	2 (6.06)
Race			*p* = 0.107	*p* = 0.991	*p* = 0.203	*p =* 0.640	*p* = 0.549	*p* = 0.215
Asian	133 (33.84)	93 (41.89)	44 (33.08)	37 (39.78)	37 (27.82)	29 (31.18)	21 (15.79)	20 (21.5)
Black	26 (6.62)	6 (2.70)	6 (23.08)	2 (33.33)	3 (11.54)	2 (33.33)	2 (7.69)	0 (0)
Latinx	130 (33.08)	64 (28.83)	51 (39.23)	23 (35.94)	44 (33.85)	14 (21.88)	25 (19.23)	5 (7.81)
White	48 (12.21)	29 (13.06)	22 (45.83)	13 (44.83)	16 (33.33)	8 (27.59)	6 (12.5)	5 (17.24)
Multiple races and other race	56 (14.25)	30 (13.51)	27 (48.21)	12 (40)	17 (30.36)	6 (20)	8 (14.29)	4 (13.33)

*p < 0.05, **p < 0.01, ***p < 0.001. T1: October 2020, T2: March 2021. Each variable may have a different total number, due to missing data.

Anxiety and depression were common in this primarily young adult population (Table 1). In October 2020, 38.07% met the criteria for anxiety and 29.85% met the criteria for depression; in March 2021, 40.65% met the criteria for anxiety and 27.57% met the criteria for depression. Suicidal ideation was also high with 15.94% of participants in October 2020 and 16.04% in March 2021 reporting suicidal thoughts. The prevalence of anxiety was higher among women compared to men in both time periods (43.94% vs. 20%, *χ*^2^ (2) = 19.1, *p* < 0.001 in October 2020 and 44.83% vs. 20.45%, *χ*^2^ (2) = 13.31, *p* = 0.003 in March 2021). Compared to heterosexual participants, non-heterosexual participants (combining gay, lesbian, bisexual, mostly heterosexual, and other) showed significantly higher prevalence in anxiety (54.41% vs. 34.78%, *χ*^2^ (1) = 9.16, *p* = 0.002), depression (55.88% vs. 24.53%, *χ*^2^ (1) = 25.91, *p* < 0.001), and suicidal ideation (33.82% vs. 11.8%, *χ*^2^ (1) = 20.02, *p* < 0.001) in October 2020. Participants aged 18–25 showed significantly higher prevalence of suicidal ideation compared to older adults (18.18% vs. 8.25%, *χ*^2^ (1) = 4.91, *p* = 0.027) in October 2020.

**Figure 1:**
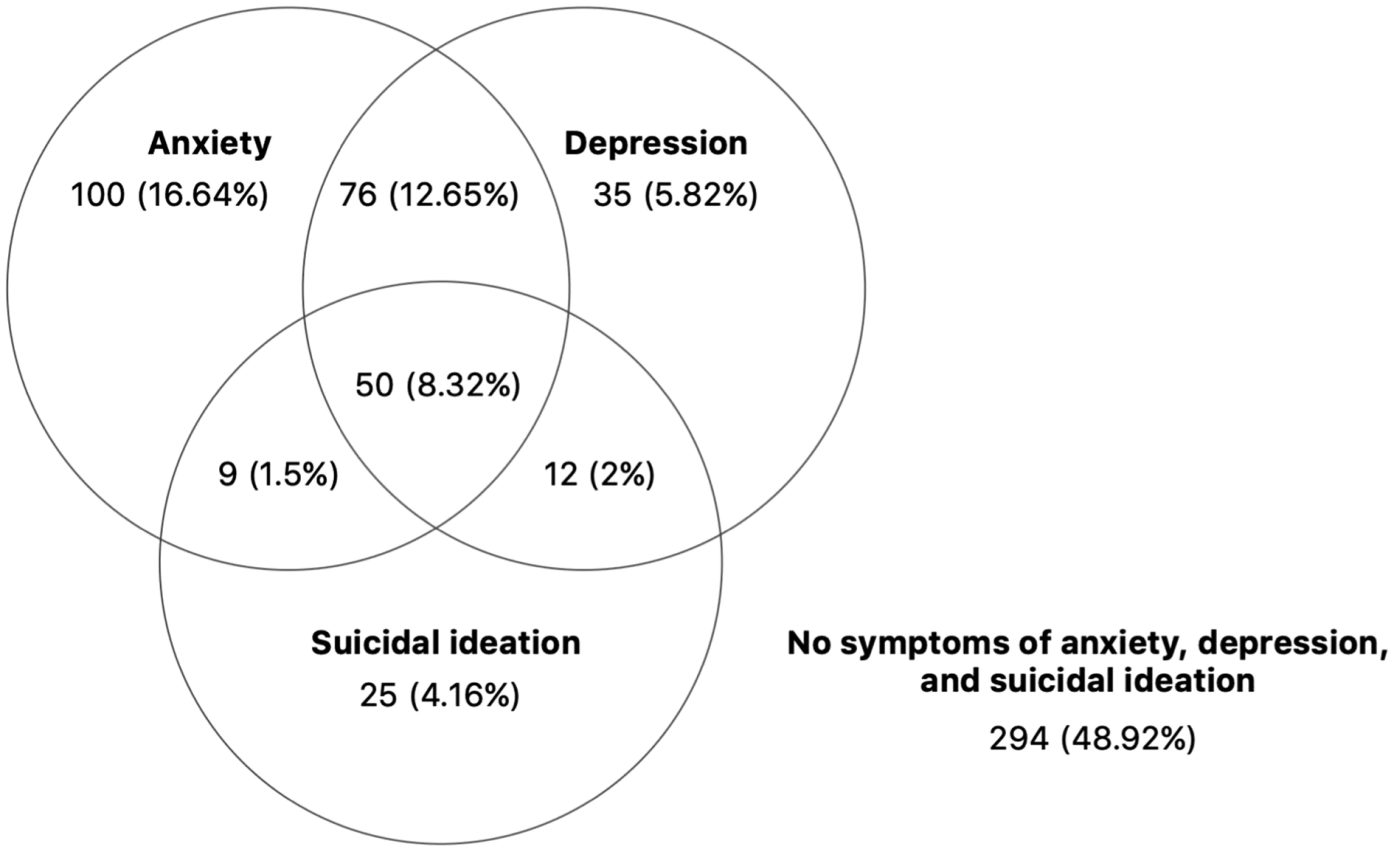
Comorbidity of anxiety, depression, and suicidal ideation. This graph displays the proportion of participants classified with each of the three mental health disorders in October 2020 and March 2021 (Total: 601).

Examining differences in mental health outcomes across the two time periods, we found that the prevalence of anxiety, depression, and suicidal ideation were not significantly different (chi-square tests, respectively, *p* = 0.533, 0.555, 0.975). We present data on the comorbidity of these mental health outcomes in Figure 1. Among participants who answered all questions in the PHQ-4 and item 9 of the PHQ-9 across the two different time periods (*N* = 601), 50 of them (8.32%) were classified as meeting the criteria for all three outcomes (Figure 1).

In addition to anxiety, depression, and suicidal ideation, the October 2020 survey assessed loneliness and coping strategies that students were employing to manage the challenges of the pandemic. More than half the participants reported loneliness for several days, more than half the days, or nearly every day (Table 2). Participants provided multiple answers for their coping strategies. The most common coping strategy was watching shows, listening to music, or playing video games (69.01%). More than half of the participants reported sleeping (56.70%), taking breaks (51.65%), connecting with friends (52.31%), and connecting with family (51.21%) to help them manage the pandemic. Other coping strategies are described in Table 2.

**Table 2:** Additional mental health survey results from October 2020 (*N* = 394) and March 2021 (*N* = 222).

October 2020 questions	*n* (%)	March 2021 questions	*n* (%)
**Loneliness**		**How mental health changed since the pandemic**	
Not at all	164 (41.94)	Worse	103 (46.40)
Several days	126 (32.34)	Same	78 (35.14)
More than half the days	52 (13.30)	Better	25 (11.26)
Nearly every day	49 (12.53)	Unsure	16 (7.21)
**Coping strategy** ^†^		**Whether mental health worsened due to the pandemic**	
Watching shows, listening to music, or playing video games	314 (69.01)	Yes	36 (35.64)
Sleeping	258 (56.70)	Somewhat	61 (60.41)
Taking breaks	235 (51.65)	No	2 (1.98)
Connecting with friends	238 (52.31)	Unsure	2 (1.98)
Connecting with family	233 (51.21)	**Distressing household experiences** ^†^	
Exercise or spending time outdoors	213 (46.81)	Experienced mental health problems	109 (43.95)
Art, music, journaling, or another creative expression	152 (33.41)	Lost a job or lost income	85 (34.27)
Taking care of a pet	141 (30.99)	Worked without PPE or social distancing	54 (21.77)
Mindfulness practice or breathing exercises	126 (27.69)	Became sick with COVID-19	48 (19.35)
Using alcohol or cannabis	106 (23.30)	Delayed necessary medical care	31 (12.50)
Using other drugs	14 (3.08)	Experienced violence or abuse	13 (5.24)
		Had to reduce work to care for children	12 (4.84)
		Was evicted, foreclosed on, or moved to save money	9 (3.63)
		**How often you are top of things**	
		Not at all	57 (26.89)
		Several days	92 (43.40)
		More than half the days	45 (21.23)
		Nearly every day	18 (8.49)

† Multiple answers were allowed.

Using a generalizable linear regression model with robust standard errors, we found that students who experienced anxiety or depression were less likely to report taking breaks (Prevalence Ratio 0.73, 95% CI 0.56, 0.97, *p* = 0.029 for anxiety and PR 0.68, 95% CI 0.49, 0.94, *p* = 0.021 for depression) than students without anxiety or depression. Students who experienced depression, suicidal ideation, or reported feeling lonely nearly every day were less likely to report connecting with family (PR 0.69, 95% CI 0.49, 0.95, *p* = 0.027 for depression; PR 0.49, 95% CI 0.30, 0.80, *p* = 0.004 for suicidal ideation; PR 0.32, 95% CI 0.15, 0.67, *p* = 0.003 for loneliness nearly every day). Those who reported feeling lonely nearly every day had more than twice the prevalence of coping by sleeping than students who did not report feeling lonely (PR 2.25, 95% CI 1.19, 4.23, *p* = 0.012).

The March 2021 survey asked participants to report on their overall mental health: almost half of the participants’ reported that their mental health had become worse since the pandemic (46.40%), with 96.05% reporting that this was due fully or partially to the COVID-19 pandemic (Table 2). Participants reported feeling on top of things for several days (43.40%), more than half the days (21.23%), and nearly every day (8.49%). Student researchers also inquired about various distressing experiences that participants or someone in their household may have had because of the COVID-19 pandemic in the March 2021 survey (Table 2). Almost half of the participants reported that they or someone in their household had experienced mental health problems (43.95%). Additional stressors reported included loss of a job or income (34.27%), working without PPE or social distancing (21.77%), becoming sick with COVID-19 (19.35%), delaying necessary medical care (12.50%), experiencing violence or abuse (5.24%), reducing work to care for children (4.84%), and experiencing eviction, foreclosure, or being required to move to save money (3.63%).

### Reflections on the research process

Through use of this participatory CURE, the student-faculty research team was able to obtain answers to original research questions of interest to the team members, campus stakeholders, and the broader public health community. Topics across the two semesters of the CURE were different, reflecting the CURE principle that new research questions and directions be generated each semester, in collaboration with the students in the course. In fact, there is an expectation in CURE research that work in a CURE is “unlikely to look the same from year to year” (27).

Students reported increased motivation to work on this participatory CURE than projects for other courses. One student reported: “*the prospect of collecting meaningful data and promoting change through our analysis motivated us to choose a topic that would interest us for the next 4+ months. We were all very interested in mental health and knew we would find shifts in wellness during COVID-19 because of our own experiences*.” Another student reflected that the participatory CURE “*allowed me the chance to practically apply concepts we were learning in class.*” The graduate research assistant shared, “*I started this work at a time where I was feeling uninspired in my internship and struggling with motivation in grad school. Collaborating with this team helped me cope with my own isolation and loneliness.*”

While highly motivating, working on research that had potential for application outside the classroom context also made students feel nervous. One student shared that she experienced imposter syndrome and felt her “*excitement being quickly overtaken by anxiousness and doubt in my capability to keep up with the rest of the team*.” The regular meeting structure, conversations with her peer co-researchers, and candid talks with the graduate student assistant working on the study all helped allay this student’s concerns. She noted that the faculty created “*an open space to speak out, for us to ask questions*” and encouraged students to “*step outside of our comfort zone*.” Another student researcher shared “*The level of professionalism required for this project was honestly such a new experience for everybody involved!*”

As others have reported, participating in a CURE made some students more interested in pursuing graduate education. One student co-researcher shared, “*I really enjoyed how the curriculum changed to reflect that semester’s cohort. Working on this study and actively exploring epidemiology made me much more interested in public health. Now I want to pursue an MPH with a concentration in epidemiology/biostatistics.*”

As with other participatory methodologies, this study made use of the participant’s lived experiences to guide the research questions, strengthening their relevance. For example, loneliness was a topic that students selected because it resonated with their own experiences. One student shared “*I already had an idea what isolation can do to a person’s mental wellbeing from my job working at a 55+ aged community for skilled nursing and memory care… I witnessed elderly residents’ mental health decline from isolation because they were unable to receive social support from their family due to the lockdown protocol…. I was also isolating myself in my room and encountering loneliness myself. When speaking to other people my age they reported they were also experiencing similar feelings. I wondered how these experiences were being felt by my own community of college students.*” Working on this topic allowed this student to draw on her professional experience, her personal experience, and her academic knowledge. Students were similarly encouraged to decide for themselves what they thought the most valuable means of disseminating the study findings would be and selected a public-facing blog where they could write findings in lay language and post videos of short professional presentations of the research findings.

As other faculty engaged in CUREs have reported, the faculty collaborating in this participatory CURE reported that it was highly satisfying (28). One faculty member shared: “*I find doing student-partnered research incredibly meaningful. I love having the opportunity to more closely integrate my teaching, research, and mentorship. While I sometimes feel like the stakes for a CURE are higher than for other teaching approaches and it requires a deeper investment of time, that additional work is offset by the joy of seeing students motivated to do work for the science itself rather than for a grade.*” The other faculty member pointed out that employing a participatory CURE approach was an effective way to “*pursue research productivity and teaching effectiveness at the same time.*”

These perspectives highlight the potential for transformative experiences for students and faculty engaged in participatory CURE, especially on topics related to adolescent mental health and wellbeing in schools and universities. The Course-Based Undergraduate Research Experiences Network (CUREnet) database provides details on 25 CUREs across 24 campuses in multiple countries; despite the diverse topics and contexts, all of the courses are in STEM fields (29). This article extends the CURE literature by providing an example of a CURE within the public health field.

## Discussion

In our study of diverse young adults, we found that 46.93% had evidence of clinical levels of either anxiety or depression. This is consistent with the Healthy Minds Study from Fall 2020, which sampled over 30,000 college students on campuses across the country and found 47% met criteria for depression and/or anxiety disorders (30). While the Healthy Minds Study used different measures for anxiety and depression than we used in our study, these data are also similar to the findings from the U.S. Census Household Pulse survey, which, like the present study, used the PHQ-4. Throughout the pandemic, the Household Pulse survey has found that young adults ages 18–24 have the highest level of anxiety and depression of any age group (31). In December, midway between our two surveys, 56.2% of 18–24-year old’s surveyed reported symptoms of anxiety and/or depression. Depression and anxiety were more common in households that had experienced job loss and among racial and ethnic minority populations (31).

The level of depression in our study was slightly higher than has been reported in this specific student population previously and higher than most prior studies of college student mental health (32), likely reflecting the increase in depression in the population during the COVID-19 pandemic (33). However, these prior studies used the PHQ-9, a different measure of depression, rather than the PHQ-4, and thus observed differences might also reflect these different scales.

Compared to the Fall 2020 Healthy Minds Study, our participants reported more suicidal ideation in both time periods (Healthy Minds found 13% past year suicidal ideation vs. 15.94% and 16.04% past 2 weeks suicidal ideation in our study) (30). Our participants reported less loneliness than students in the Healthy Minds Study (41.94% of participants in our study reported not feeling lonely at all vs. 34% of students in the Healthy Minds Study reported feeling isolated from others hardly ever) (30). Suicidal ideation and loneliness are critical factors to track as even before the pandemic, suicide was the second leading cause of death in young people and the social isolation brought on by the pandemic is expected to exacerbate this problem (34).

Similar to surveys of Canadian students in the early months of the pandemic, over half of students in our October 2020 survey reported connecting with family or friends to help them cope with the pandemic and students who used these social strategies had better mental health (35). While a similar proportion of participants also reported using exercise to cope (our study: 46.81% vs. Canadian study 54.5%), twice as many participants in our study used mindfulness (27.69%) compared to students in the Canadian study (12.0%). More than half of our participants reported sleeping to cope, compared to just 17.5% of Canadian students.

The adverse mental health impacts of the COVID-19 pandemic are likely to be felt for years after the pandemic ends (36). Recognizing the extensive mental health challenges faced by young adult college students, we suggest that Universities proactively employ universal approaches to improving mental health, rather than relying on counseling and psychological services within health centers to treat all students who could potentially benefit from mental health care (37). Universal approaches target mental health interventions to all students through multiple, overlapping strategies rather than rely on the typical client/therapist mental health care model. Such approaches might focus on building the skills of resilience, which research shows can be actively taught (38). To combat loneliness and improve general emotional wellbeing, universities might also consider programming specifically aimed at reducing loneliness, including pedagogy training for faculty on teaching strategies that promote connections between students (39).

### Strengths

The strengths of this study include its participatory design, use of valid and reliable scales in addition to novel constructs, and good sample sizes for the research questions examined. The study quality benefits from the strong voice of students, members of the population under study, and the collaborative nature of the study. Survey questions reflected students’ interests, representing community members’ interests as well. From the students’ perspective, this study closed the gap between knowledge learned in the course and actual research and application. From the course instructors’ perspective, the CURE synergizes teaching and research, and require high commitment, creativity, investigativeness, and critical analysis (40).

### Limitations

The study findings are limited by the non-probability sampling approach, decreasing generalizability. As students in the CURE were public health majors and minors, it is likely that the study sample overrepresented students in this field of study compared to other disciplines. Students were also mostly juniors and seniors, who might have different experiences than first-year students (41). However, our empirical findings are very similar to contemporaneous larger studies, such as the non-representative Healthy Minds Study and the nationally representative Household Pulse Survey. Regarding data from two time periods, the study represents a repeated cross-sectional survey rather than a longitudinal design and so changes in variables do not reflect changes at the individual level, but rather at the population level. Although the respondents of the first survey in October 2020 were not the same respondents of the second survey in March 2021 survey, the two samples were drawn from overlapping source populations and by comparing results in the two time periods, we could observe how mental health among this college student population changed. In addition, we did not specify *a priori* how we would evaluate the process or outcomes related to using this participatory CURE approach. Future participatory CURE studies would be strengthened from *a priori* specification of the design to assess process and CURE outcomes such as student motivation and graduate school intentions.

## Conclusion

Course-Based Undergraduate Research Experiences are ripe locations for integrating participatory research approaches, such as Community-Based Participatory Research. This participatory CURE gave rise to a deeper understanding of college students’ mental health burden and highlighted both areas of risk and factors that are protective for this population. Students who engage in CUREs can be strong advocates for the application of research findings, bringing their youthful passion to solve complex problems. In the case of these findings, we hope that colleges and universities take seriously their obligation to serve and protect their student population by increasing mental health services, given the high need in this young adult population. While CUREs have been slow to be adopted outside of STEM fields, this study adds to a growing body of literature demonstrating the feasibility of CUREs in public health and other social sciences.

## Data availability statement

The datasets presented in this article are not readily available because of concerns that privacy of research participants may be compromised when variables are combined. Requests to access the datasets should be directed to miranda.worthen@sjsu.edu.

## Ethics statement

The studies involving human participants were reviewed and approved by San José State University IRB approval (IRB protocol tracking numbers 20251 and 21069). Exempt registration was received. Waiver of signed consent was approved. Written informed consent for participation was not required for this study in accordance with the national legislation and the institutional requirements.

## Author contributions

MW developed the CURE and taught the course. KN, DP, HM, TA, and the PH 161 cohorts of Fall 2020 and Spring 2021 designed the study and collected the data. CP, MMF, TME, KN, DP, HM, TA, and MW conducted initial data analysis. CP further analyzed the data and produced the tables and figures. CP, MW, KN, TME, and MMF conceived of the manuscript. MW drafted the manuscript, with contributions from KN, TME, CP, and MMF. All authors contributed to the article and approved the submitted version.

## Funding

This work was supported by the Thoracic Foundation.

## Conflict of interest

The authors declare that the research was conducted in the absence of any commercial or financial relationships that could be construed as a potential conflict of interest.

## Publisher’s note

All claims expressed in this article are solely those of the authors and do not necessarily represent those of their affiliated organizations, or those of the publisher, the editors and the reviewers. Any product that may be evaluated in this article, or claim that may be made by its manufacturer, is not guaranteed or endorsed by the publisher.

## Footnotes

^1^ https://wellnessduringcovid.weebly.com/
